# Combining albumin deficiency and acute exercise reduces hepatic lipid droplet size in mice

**DOI:** 10.1186/s12944-023-01845-9

**Published:** 2023-06-21

**Authors:** Yi Zhang, Mirandia Szramowski, Shuhan Sun, Gregory C. Henderson

**Affiliations:** Department of Nutrition Science, Purdue University, 700 Mitch Daniels Blvd., West Lafayette, IN 47907 USA

## Abstract

Hepatic lipid droplets (LDs) are implicated in ectopic lipid accumulation. The core of LDs, triacylglycerol (TAG), is synthesized from the esterification of fatty acids to a glycerol-3-phosphate (G-3-P) backbone. Albumin transports plasma free fatty acids, and previously albumin knockout (Alb^−/−^) mice were shown to exhibit lower hepatic TAG levels than wildtype (WT). Exercise is a beneficial strategy to alter hepatic metabolism, but its impacts on reducing hepatic lipids are far from satisfactory. The aim of this study was to investigate the combined effect of albumin deficiency and acute exercise on hepatic LDs. Eight-week-old male Alb^−/−^ and WT mice were divided into sedentary and exercise groups. Exercised mice performed a 30-min high-intensity exercise bout. Results showed that sedentary Alb^−/−^ mice had smaller hepatic LDs (*P* < 0.0001), associated with mitochondria, while WT mice exhibited larger LDs, surrounded by glycogen granules. Following acute exercise, hepatic LDs in Alb^−/−^ mice reduced by 40% in size, while in WT increased by 14% (*P* < 0.0001). The maintenance of WT hepatic LDs was associated with elevated G-3-P level (*P* < 0.05), potentially derived from glycogen (R = -0.32, %change in glycogen versus LD content, *P* < 0.05). The reduction in Alb^−/−^ mice LDs after exercise was possibly due to their low glycogen level. In conclusion, Alb^−/−^ mice exhibited an enhanced capacity for reducing hepatic LD size and content in response to exercise. These findings suggest that modulating albumin’s functions combined with exercise could be a potential strategy to reduce ectopic lipid deposition in the liver.

## Introduction

The majority of lipid in the body is stored in adipose tissue [1], yet still ectopic lipid deposition occurs in other organs, such as muscle and liver. Abnormal lipid accumulation in the liver is a risk factor for developing nonalcoholic fatty liver diseases (NAFLD) [2, 3] and is involved in the development of other systemic abnormalities, including type 2 diabetes mellitus, chronic kidney disease and cardiovascular diseases [4]. Hepatic lipid accumulation occurs through biogenesis and expansion of lipid droplets (LDs), the lipid storage organelle of the cell [5].

LDs are a newly described organelle and the understanding of their size, structure and function is far from being complete [6]. The core of LDs contains triacylglycerol (TAG) and other neutral lipids, including cholesteryl esters and retinyl esters [7]. LD biogenesis is mainly regulated by the synthesis of TAG [8]. The majority of TAG is formed through the glycerol-3-phosphate (G-3-P) pathway, which is characterized by the esterification of fatty acids to a G-3-P backbone [9]. Two sources of G-3-P are glycerol and dihydroxyacetone phosphate (DHAP). DHAP is an intermediate in the glycolysis pathway and a precursor for G-3-P production. Emerging evidence has indicated LDs as more than just a lipid storage depot but are highly dynamic organelles that mediate various metabolic processes. These processes include protecting cells from lipotoxicity via sequestering free fatty acid (FFA), and protecting against endoplasmic reticulum (ER) stress and mitochondrial damage [6]. However, an excessive abundance of LDs can be problematic. Dysregulation in LD homeostasis generates larger LDs which are involved in the initial events in NAFLD pathogenesis [10]. A major source of LD formation is FFA released from adipose tissue, which is elevated in obesity or when demands for fat metabolism are increased during fasting [11]. Other sources include dietary fat from chylomicrons in the post-prandial state and de novo lipogenesis [12]. There could be different strategies for preventing hepatic lipid accumulation. One approach would be to diminish the amount of substrate supply by reducing the FFA release from adipose tissue.

Plasma FFA level is recognized as a risk factor for metabolic disorders [11]. Elevated plasma FFA was found in patients with NAFLD and was identified as a predictive factor for advanced fibrosis [13]. Pharmacological strategies to lower plasma FFA and reduce uptake into tissues encompass niacin treatment, as well as antagonists of adipose triglyceride lipase (ATGL) and endothelial fatty acid transporters [14–16], but no such approach has become approved for effective treatment of ectopic lipid deposition. One novel method of lowering plasma FFA is to target albumin, the main binding protein of FFA in plasma [17]. Alb^−/−^ mice were previously shown to exhibit low levels of plasma FFA and low hepatic TAG, but the LD characteristics in the liver have not yet been investigated. Furthermore, it was not yet known how this mouse model responds to healthful lifestyle factors such as exercise.

Physical activities like aerobic or resistance exercise are shown to reduce intrahepatic lipids, especially in patients with NAFLD [18]. However, currently, the typical 10–30% reduction of liver steatosis with regular exercise is disappointingly modest [19–22]. Also, the type of exercise exerts different effects on the participants. Moderate-intensity of aerobic exercise requires a high cardiorespiratory demand and is often associated with low pleasure and adherence [23]. Previous studies suggested that high-intensity interval exercise (HIIE) is more well-accepted and sustainable [22]. While there may be various genetic mouse models exhibiting improvements in health, it is of interest to understand how mouse models respond to stressors in order to improve knowledge of metabolic regulation. HIIE was chosen as a healthful metabolic stressor and applied to a gene knockout mouse model (albumin deficient mice) that was previously shown to have a propensity for exhibiting favorable metabolic health. The aim was to investigate the combined effect of HIIE and albumin deficiency on hepatic LDs, including their abundance, morphology, and localization near glycogen and mitochondria. It was hypothesized that Alb^−/−^ mice would have smaller and fewer hepatic LDs compared to those in WT during resting. It was also reasoned that in response to exercise, LDs in Alb^−/−^ mice would undergo different morphological changes than WT mice. Taken together, the combined effect of albumin deficiency and acute exercise were investigated in order to shed light on potential strategies for reducing ectopic lipid deposition in the liver.

## Methods

### Animals

All experiments were approved by the Purdue University Animal Care and Use Committee. Male Alb^−/−^ mice and wildtype (WT) littermates were studied (*n* = 12 per genotype). The mice were derived from a breeding colony at Purdue University. Genotyping of the mice was conducted by endpoint PCR. Original breeders (C57BL/6 J-Albem8Mvw/MvwJ, Strain #025200) were purchased from the Jackson Laboratory (Bar Harbor, ME). This whole-body knockout model was achieved by TALEN-mediated deletion in Alb gene, resulting in the absence of any albumin protein in the blood [24]. Mice were housed in individually ventilated cages with up to five mice per cage after weaning (3-week-old) and then were singly housed when they reached 6 weeks old. The housing was kept at a temperature of around 22 °C, humidity of 30–70%, 61 air exchange per hour in the cages, and a 12 h light/dark photoperiod with lights on at 6 A.M.. All the mice were allowed ad libitum access to water and food (Teklad Global 18% Protein Rodent 2018S; Envigo, IN, USA) after weaning at 3 weeks of age for their lifetimes. All mice were euthanized at 8 weeks of age.

### Body composition analysis

All mice were subjected to analysis of body composition and total body water by magnetic resonance (EchoMRI LLC, Houston, TX) at the age of 6 weeks old in the unanesthetized state. Before the scanning of mice, the instrument was calibrated according to manufacturer instructions. Each mouse was placed in a plastic tube which was inserted into the instrument for scanning. Two scans were performed for each mouse and results were averaged. Mice were immediately returned to their cages following the scans.

### Incremental exercise performance test

To test exercise capacity, an incremental exercise test was conducted using a rodent treadmill (Columbus Instruments, Columbus, OH, USA). All mice, at the age of 7 weeks, were acclimatized to the treadmill for two consecutive days. The acclimatization consisted of 5 m/min walking for 5 min at 0° incline. The day after the second acclimatization, mice were subjected to the incremental test. All 24 mice were fasted for 2.5 h before the test. For the incremental test, the incline was set up as 25° and the speed automatically increased by 1.5 m/min, following the warmup period of 5 min at 6 m/min. The test ended when mice reached exhaustion, defined by when they remained on the shock grid for 5 s; this time duration was recorded for each mouse to indicate their performance. The shock grid was set to an intensity value of 1 (arbitrary units) and frequency of 1 Hz.

### Insulin tolerance test

To test the insulin sensitivity in mice after exercise, each genotype was randomly divided into the sedentary control group (CON, *n* = 6) or the exercise group (EX, *n* = 6). The mice were ~ 7 weeks old at the time of the insulin tolerance test. Food was withdrawn 2.5 h before exercise or the exact time of day in the sedentary control mice. The body weights of the mice were collected. The treadmill incline and shock grid settings were as described above for the incremental exercise performance test. The HIIE process started with a 5-min warmup at 5 m/min followed by a 30-min HIIE bout. After the warmup, mice underwent repeated cycles of 30-s sprinting followed by 1-min walking, as described previously [25, 26]. There was a total of 20 sprints. The sprinting speed started at 15 m/min on the first cycle and increased by 5 m/min every cycle until reaching 30 m/min for each of the remaining sprint intervals in the 30-min exercise bout. After exercise, all mice were rested without food for 1 h and then injected with insulin (Novolin-R, Novo Nordisk, Denmark) at 0.45U/kg body weight. At time points of 0, 20, 40, 60, 80, 100, and 120 min, blood was taken from the tail and blood glucose concentration was measured by a point-of-care device (Prodigy, Charlotte, NC, USA) in duplicate and then averaged. Area under the curve (AUC) was calculated as a Riemann Sum (i.e., by the trapezoidal rule).

### Standardized exercise bout and tissue collection

To test the responses to a challenging exercise session, the mice at the age of ~ 8 weeks were subjected to the same bout of HIIE as described above. For each genotype, mice were assigned to the CON group (*n* = 6) or the EX group (*n* = 6). Food was withdrawn 2.5 h before exercise or the exact time of day in the sedentary control mice. Immediately after HIIE, mice from the exercise group were euthanized with carbon dioxide and control mice were also euthanized with the same method at the same time point. Blood was collected using cardiac puncture and then livers and soleus muscle were collected rapidly. A portion of the liver and the entirety of the soleus muscle was fixed in fixative buffer (2.5% glutaraldehyde, 1.5% paraformaldehyde in 0.1 M sodium cacodylate buffer), and the remaining portion of the liver was immediately frozen in liquid nitrogen and then stored at -80 °C until further analysis. Muscle samples were collected for comparison to provide context for electron microscopy findings on the liver.

### Plasma biochemical measurement

After euthanasia, blood was collected using cardiac puncture and plasma was separated via centrifuge. Plasma FFA levels were measured using an FFA kit from Millipore-Sigma. Plasma glycerol and TAG were measured using free glycerol reagent (Sigma-Aldrich) and triglyceride reagent (Sigma-Aldrich).

### Coomassie gel staining

Albumin deficiency was previously demonstrated in Alb^−/−^ mice through mass spectrometry analysis [17]. To confirm this expectation in the present study, SDS-PAGE was used. Plasma samples were diluted with gel loading buffer containing Laemmli Sample Buffer (Biorad, Hercules, CA, USA) and β-mercaptoethanol. Plasma proteins were then separated by 4–15% Tris–HCl precast gel (BioRad), with the human albumin standard as a reference. After that, the gel was stained by Coomassie Brilliant Blue R-250 Staining Solution (Biorad) for one hour, followed by destaining with Coomassie Brilliant Blue R-250 Destaining Solution (Biorad). The resulting gel was then scanned using an Odyssey CLx Imager (LI-COR Biosciences, Lincoln NE, USA).

### Biochemical assays

To quantify the content of TAG in liver, 20–30 mg sample from each mouse was homogenized in a mixture of heptane, isopropanol, and water by a Tissuelyzer bead homogenizer (Qiagen, Germantown, MD, USA), using the method previously described [17] and a commercially available kit for detection (Millipore-Sigma). Using the kit, TAG was digested by lipoprotein lipase, liberating free glycerol. The level of free glycerol was determined by colorimetric analysis against the standard curves built from a standard glycerol solution. The concentration of hepatic glycogen was obtained with a commercially available glycogen assay (Millipore-Sigma) according to the manufacturer’s instructions. The concentration of G-3-P in liver was also assessed with a kit (Abcam, Cambridge, UK).

### Transmission electron microscopy (TEM) analysis

To visualize the size, volume and distribution of organelles in the liver and soleus, TEM analysis was conducted. Samples were rinsed in a fixative buffer four times, fixed in 1% osmium tetroxide and 0.8% ferricyanide. Then, they were washed in deionized water, followed by fixation in 1% uranyl acetate. After a second rinse with deionized water, the samples were dehydrated with a series of ethanol/acetonitrile steps with a transition in acetonitrile. After that, infiltration was performed using propylene oxide and resin. In the end, samples were embedded (Embed 812: DDSA: NMA 5:4:2; 0.22 DMP-30). Stained sections were visualized with a Tecnai T12 transmission electron microscope (FEI company, Hillsboro OR, USA) at 120 kV. Gatan imaging system (Gatan, Inc., Pleasanton CA, USA.) was used to capture images. Glycogen, mitochondria, and LDs were quantified by ImageJ software (Scion Image, National Institutes of Health, Bethesda, MD, USA). 12–14 images under 6,000X magnification were randomly taken from 4 mice per group for analysis. The area density of hepatic glycogen was assessed using point counting [27] with the grid size of 632 nm × 632 nm, corresponding to 0.4 μm^2^. The area density, size, and number of LDs was assessed without a grid. The criteria for LD identification include a clear monolayer or fuzzy border, a grey or white colored core and a near-circular shape. Glycogen granules in liver were identified as dark, roughly circular granule clusters and the findings for glycogen abundance by TEM were confirmed by a biochemical assay which was in agreement. Glycogen granules were not visualized in the soleus because their smaller size and abundance in this tissue make it far more challenging to identify them confidently in muscle. Two investigators were involved and were blinded to the sample information. The percentage of LD perimeter in contact with glycogen or mitochondria was quantitated by the analysts identifying the sections of LD perimeter in contact with glycogen granules or mitochondria.

### Western blot

Liver tissue was homogenized with a Tissuelyzer (Qiagen) in cell lysis buffer (Cell Signaling Technology, Danvers MA, USA) and protease inhibitor (Roche, Basel, Switzerland) and phosphatase inhibitor (Thermofisher, MA, USA). Proteins were separated by 4–15% Tris–HCl precast gel (BioRad) and then transferred onto a nitrocellulose membrane (BioRad). After drying for an hour, the membrane was blocked for another hour using 5% nonfat-dried milk in 1X TBS (Biorad). Then, the membrane was probed with the primary antibody in TBST at 4 °C overnight, followed by an additional 3 h at room temperature. After washing with TBST three times, the membrane was probed with secondary antibodies for one hour, followed by washing three times. In the end, the membrane was visualized using Odyssey CLx Imager (LI-COR Biosciences). The following antibodies were used for probing: anti-acyl-CoA dehydrogenase long chain antibody (ACADL; 1:1000; Abcam, ab128566), anti-glycerol-3-phosphate dehydrogenase 1 antibody (GPD-1; 1:1000; Thermofisher, PA5-31051), anti-ATP synthase F1 subunit α antibody (ATP5a1; 1:1000; Thermofisher, 43–9800), anti-ꞵ-actin antibody (1:1000; Cell Signaling, 4967S), IRDye® 800CW Goat anti-Mouse IgG Secondary Antibody (1:15,000; Licor, 926–32,210), IRDye® 680RD Goat anti-Rabbit IgG Secondary Antibody (1:15,000; Licor, 926–68071), IRDye® 680RD Goat anti-Mouse IgG Secondary Antibody (1:15,000; Licor, 926–68070), IRDye® 800CW Goat anti-Rabbit IgG Secondary Antibody (1:15,000; Licor, 926–32211).

### Real time PCR (RT-PCR)

Liver RNA was extracted using Rneasy Mini Kit (Qiagen) according to the manufacturer’s instructions. cDNA was made using Affinity Script QPCR cDNA Synthesis Kit (600559, Agilent Technologies, Santa Clara, CA, USA). The following TaqMan primers and probes (Thermofisher) were used for gene expression quantification. ACADL (Mm00599660_m1, 4331182), GPD-1 (Mm00515846_m1cytosolic, 4331182), Carnitine palmitoyltransferase-Iα (CPT-1α; Mm01231183_m1, 4331182), Diacylglycerol O-acyltransferase 1 (DGAT1; Mm00515643_m1, 4331182), DGAT-2 (Mm00499536_m1, Thermofisher, 4331182), Glycerol-3-phosphate acyltransferase 4 (GPAT4; Mm00497622_m1, 4331182), GPAT1 (Mm00833328_m1, 4331182), Acyl-CoA Oxidase 1 (ACOX1; Mm01246834_m1, 4331182), fatty acid synthase (FAS; Mm01204974_m1, 4331182), Citrate synthase (CS; Mm00466043_m1, 4331182), Perilipin 2 (PLIN2; Mm00475794_m1, 4331182), and 18S (Hs99999901_s1, Thermofisher, 4448484).

### Statistical analysis

Data are presented as means ± S.E.M. The results were analyzed by two-way analysis of variance (ANOVA), with genotype (WT versus Alb^−/−^) and exercise (CON versus EX) as the factors. ANOVA was followed by Fisher’s least significant difference post hoc test when appropriate. To directly probe exercise responses, a priori planned comparisons by t-test were also conducted to compare CON to EX groups within each genotype. Statistical analyses were performed with JMP version 16 (SAS Institute Inc.) with* P* ≤ 0.05 considered statistically significant.

## Results

### Alb^−/−^ mice have similar exercise capacity as WT mice despite low plasma FFA level

**Fig. 1:**
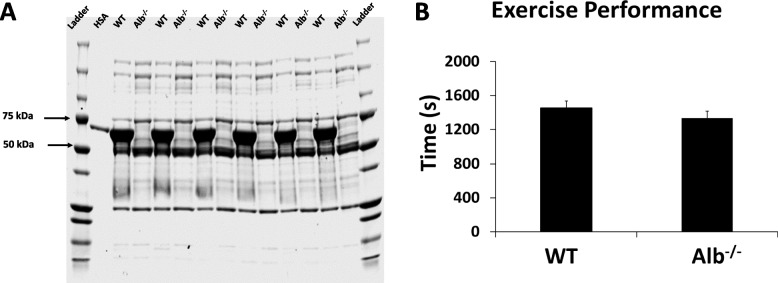
Plasma albumin and exercise capacity. **A** The absence of albumin in Alb^−/−^ mice was confirmed by Coomassie gel using human serum albumin (HSA) as a reference. **B** Exercise capacity. Analysis by t-test, *n* = 12 per group. Data are presented as the mean ± S.E.M

Firstly, the absence of albumin in Alb^−/−^ mice was confirmed by SDS-PAGE with Coomassie staining using a human albumin standard and molecular weight markers as the references. As shown in Fig. 1A, the abundance of protein at the molecular weight of albumin (66.5 kDa) was drastically reduced in Alb^−/−^ mice, while a dark band at albumin’s molecular weight was present in WT mice; this finding is consistent with previous determinations by mass spectrometry that albumin expression is essentially absent in Alb^−/−^ mice [17]. Body composition analysis was performed for all the mice at the age of 6 weeks old (Table 1), and there were no significant differences between groups. As albumin aids in the solubility of FFA in plasma, lacking albumin should result in lower plasma FFA levels. Consistent with the hypothesis, Alb^−/−^ mice had significantly lower plasma FFA levels than WT mice (282.11 ± 10.86 versus 437.62 ± 37.01 μM, *P* = 0.01). As albumin deficiency would be a metabolic challenge that could potentially impair exercise capacity, exercise capacity was tested in Alb^−/−^ and WT mice. The exercise capacity was not significantly different between genotypes (Fig. 1B, *P* = 0.23). Therefore, it was deemed appropriate to challenge WT and Alb^−/−^ with similarly challenging exercise intensity in this study of hepatic metabolism.

**Table 1:** Body composition

Genotype	Body weight (g)	Fat mass (g)	Fat-free mass (g)	Body fat percentage (%)	Total body water (g)
Wildtype	20.76 ± 0.47	1.24 ± 0.07	19.52 ± 0.44	5.94 ± 0.26	15.90 ± 0.34
Alb^−/−^	20.88 ± 0.69	1.43 ± 0.11	19.45 ± 0.62	6.78 ± 0.37	15.72 ± 0.49

Body composition analysis by magnetic resonance at 6 weeks of age. Data are presented as the mean ± S.E.M. No significant differences between groups. Alb^−/−^, albumin knockout

### Alb^−/−^ have improved insulin sensitivity, lower hepatic glycogen and hepatic lipid content than WT mice

**Fig. 2:**
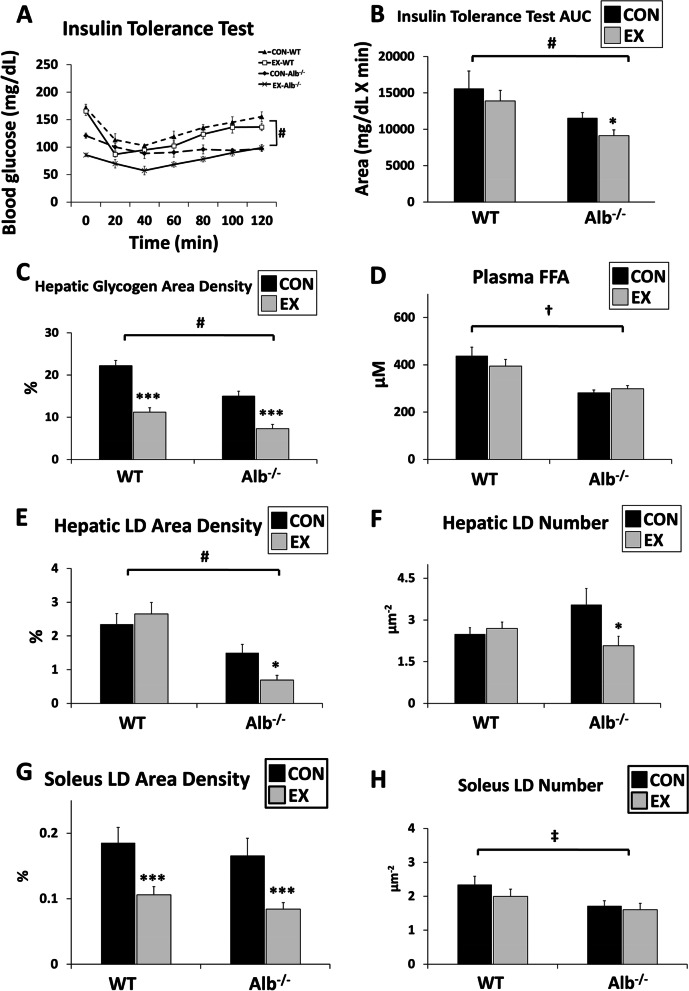
Insulin sensitivity, hepatic lipid and glycogen, muscle lipid analysis. **A** Insulin tolerance test. **B** Insulin tolerance test area under the curve (AUC). **C** Hepatic glycogen area density measured in transmission electron microscopy (TEM) images. **D** Plasma FFA level. **E** Hepatic lipid droplet (LD) area density (total abundance) measured in TEM images. **F** Hepatic LD number counted in TEM images. **G** Soleus LD area density measured in TEM images. **H** Soleus LD number counted in TEM images. EX: exercise, CON: control. Analysis by ANOVA. *n* = 6 per group. Data are presented as the mean ± S.E.M. Main effect of genotype, ^#^*P* < 0.0001, ^†^*P* < 0.01, ^‡^*P* < 0.05. EX different from CON within a genotype, *** *P* < 0.001, * *P* < 0.05

As Alb^−/−^ mice exhibited a lower plasma FFA level, it was hypothesized that they would have low levels of hepatic lipid. A lower hepatic glycogen concentration was also expected because of a need to shift to glycogen utilization when lipid supply is low. To test the hypotheses about hepatic carbohydrate and fat metabolism, insulin tolerance tests were conducted (Fig. 2A-B). There was significantly higher insulin sensitivity in Alb^−/−^ mice compared to WT (Fig. 2A, main effect of genotype, *P* < 0.0001) and an exercise-induced improvement in insulin sensitivity in Alb^−/−^ mice (Fig. 2B, *P* < 0.05). Moreover, the hepatic glycogen content was assessed using both area density measurement in TEM and a biochemical glycogen assay. TEM analysis showed nearly halved hepatic glycogen level in Alb^−/−^ mice compared to that in WT (Fig. 2C, main effect of genotype, *P* < 0.0001). Exercise in both genotypes led to a ~ 50% reduction in hepatic glycogen concentration (main effect of exercise, *P* < 0.001). Biochemical glycogen analysis results agreed with this pattern of group differences (WT CON, 28.2 ± 4.4; WT EX, 14.0 ± 2.1; Alb^−/−^ CON, 17.5 ± 1.3; Alb^−/−^ EX, 7.6 ± 2.5 μg/mg, main effect of genotype, *P* < 0.01, main effect of exercise, *P* < 0.0001). Plasma FFA levels in each group are shown in Fig. 2D, and there was no significant difference between exercise and control treatments. As expected, plasma FFA was significantly lower in Alb^−/−^ than WT (*P* = 0.01). Hepatic lipid content was assessed using TEM measurements of LDs and a biochemical TAG assay. To assess the hepatic lipid content under TEM in more detail, the LD area density and number were measured. Alb^−/−^ mice showed significantly smaller hepatic LD area than WT mice (Fig. 2E, main effect of genotype, *P* < 0.0001). For Alb^−/−^ mice, a 54% reduction was observed in LD area after exercise (*P* < 0.001) but no significant change with exercise in WT. Also, the LD number was reduced in the Alb^−/−^ but not WT mice with exercise (Fig. 2F, *P* < 0.05). For TAG concentration by biochemical analysis of the liver, there was a significant main effect of genotype only (*P* < 0.01) where Alb^−/−^ mice contained lower TAG in both groups (WT CON, 10,423 ± 1,316; WT EX, 11,534 ± 899; Alb^−/−^ CON, 7,297 ± 1,202; Alb^−/−^ EX, 7,378 ± 811 nmol/g). TEM analysis for soleus showed an exercise effect on both genotypes with approximately 50% reduction in LD area density (Fig. 2G, main exercise effect,* P* < 0.001). Moreover, Alb^−/−^ mice contained lower LD numbers in soleus (Fig. 2H, main genotype effect,* P* < 0.05). Unlike the liver, for muscle, the responses in the LD area and number to exercise did not depend upon genotype.

### Alb^−/−^ mice have smaller hepatic LDs and their LDs shrink after exercise

**Fig. 3:**
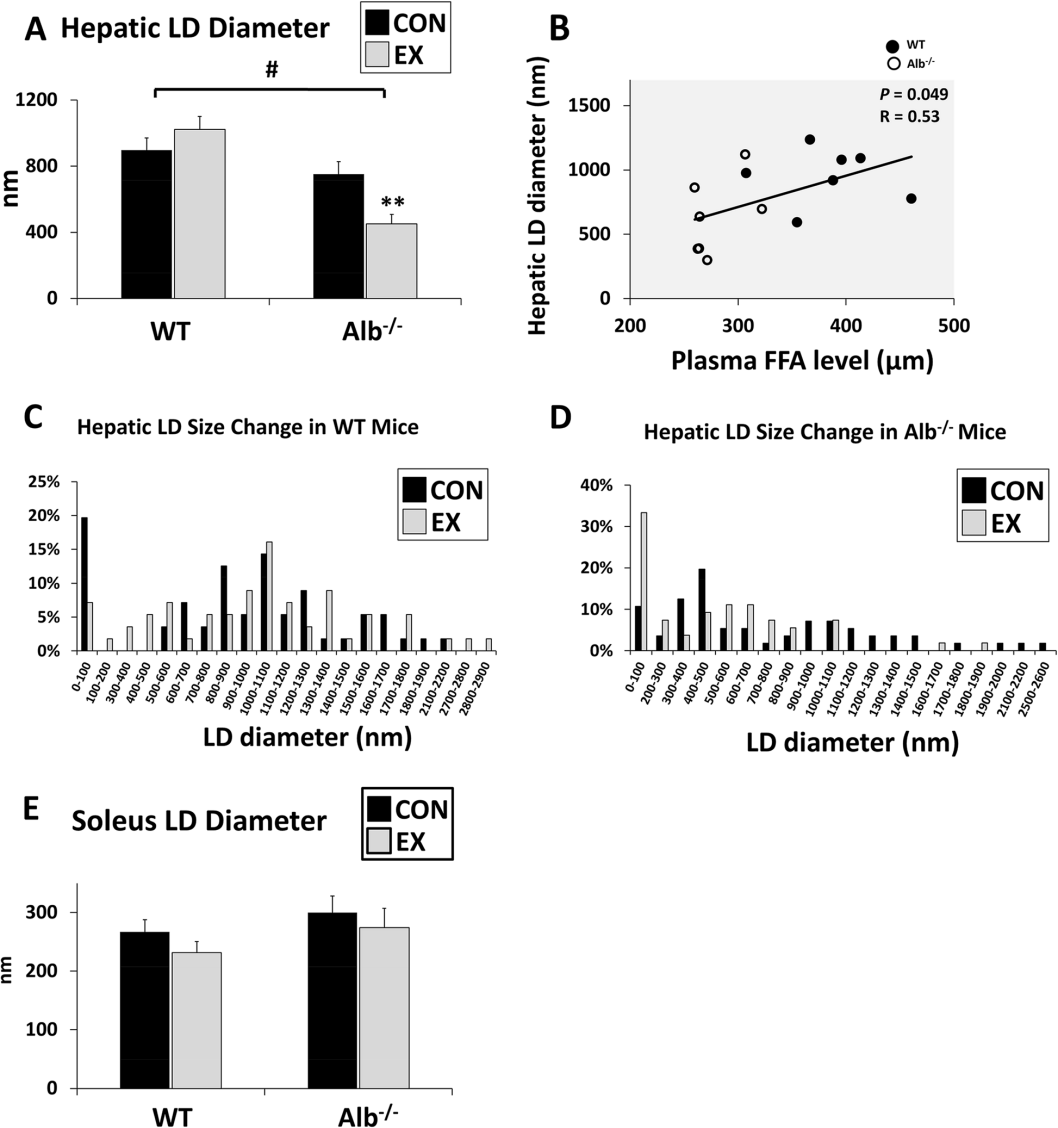
Lipid droplet (LD) size. **A** Hepatic LD diameter measured in transmission electron microscopy (TEM) images. **B** Positive correlation between plasma free fatty acid (FFA) level and hepatic LD diameter in all 24 mice. R = 0.53 (*P* < 0.05). **C** Hepatic LD size change in WT mice. After exercise, LDs tended to grow bigger, shifting the distribution to the right. **D** Hepatic LD size change in Alb^−/−^ mice. After exercise, LDs tended to become smaller, shifting the distribution to the left. **E** Soleus LD diameter measured in TEM images. EX: exercise, CON: control. Analysis by ANOVA. *n* = 6 per group. Data are presented as the mean ± S.E.M. Main effect of genotype, ^#^*P* < 0.0001. EX different from CON within a genotype, ** *P* < 0.01

To understand hepatic LD features in different groups, the diameters of the LDs were measured. It was found that LDs were significantly smaller in Alb^−/−^ mice than in WT mice (Fig. 3A, main effect of genotype, *P* < 0.0001). Importantly, in Alb^−/−^ mice, LDs then further reduced by 40% in size after exercise (compared with + 14% in WT, *P* < 0.0001). Moreover, the LD size was positively correlated with plasma FFA concentration (Fig. 3B, *R* = 0.53, *P* < 0.05). The number of LDs was also counted within different diameter ranges in each group (Fig. 3C-D). The graphs showed a slight right-shifted distribution in WT mice after exercise, indicating maintenance of LD size with a slight tendency toward expansion. On the contrary, the distribution in Alb^−/−^ mice shifted to smaller diameters on the left of the axis, showing a shrinking of LDs after exercise. In contrast to the liver, soleus exhibited no difference in the size of LDs between genotypes and treatment groups (Fig. 3E).

### LDs in Alb^−/−^ mice are more locally associated with mitochondria and less with glycogen

**Fig. 4:**
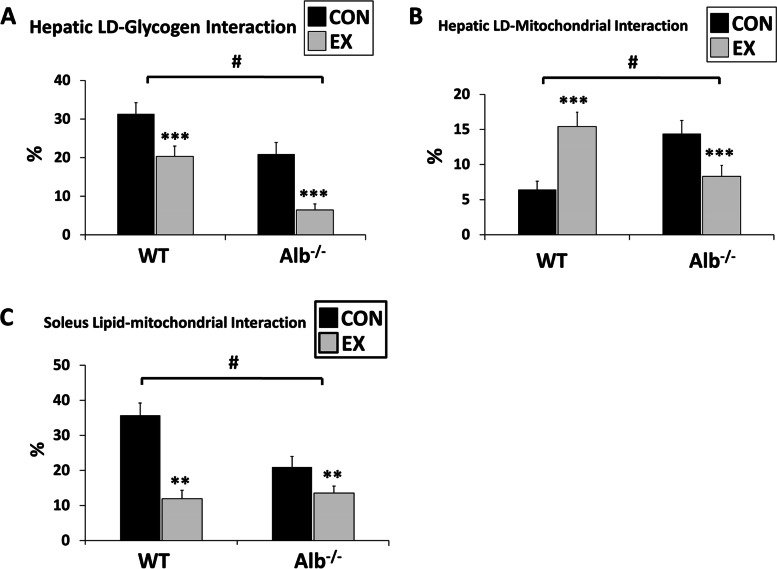
Lipid droplet (LD)’s association with mitochondria and glycogen. **A** Hepatic LD and glycogen interaction measured in transmission electron microscopy (TEM) images. The interaction was assessed by the percentage area overlapped in LDs and glycogen. **B** Hepatic LD and mitochondria interaction measured in transmission electron microscopy (TEM) images. **C** Soleus LD and mitochondria interaction measured in TEM images. The interaction was assessed by the percentage area overlapped in LDs and mitochondria. EX: exercise, CON: control. Analysis by ANOVA. *n* = 6 per group. Data are presented as the mean ± S.E.M. Main effect of genotype, ^#^*P* < 0.0001. EX different from CON within a genotype, *** *P* < 0.001, ** *P* < 0.01

In an effort to reveal the interaction between LD and glycogen as well as mitochondria, measurements were conducted in the percentage area overlapped in LD and glycogen as well LD and mitochondria in liver. For LD-glycogen interaction in liver, Alb^−/−^ mice showed a significantly lower level than WT mice (main effect of genotype, *P* < 0.0001; Fig. 4A). Both genotypes experienced a reduced lipid-glycogen interaction in the exercise group compared to control (main effect of exercise, *P* < 0.0001). Notably, Alb^−/−^ mice showed a significantly larger relative reduction in LD-glycogen interaction after exercise in the liver (percentage change: -69% versus -34%, *P* < 0.01). For lipid-mitochondrial interaction in the liver, two-way ANOVA results revealed a significant interaction between genotype and group (Fig. 4B, *P* < 0.0001). Alb^−/−^ exercise showed 42% reduction to the control group, while WT exercise had a 105% increase compared to the control group (*P* < 0.0001). In the soleus, LD-mitochondrial interaction experienced a significant difference between genotypes (Fig. 4C, main effect of genotype, *P* < 0.0001) and a reduction after exercise in both WT and Alb^−/−^ mice (main effect of exercise,* P* < 0.01).

**Fig. 5:**
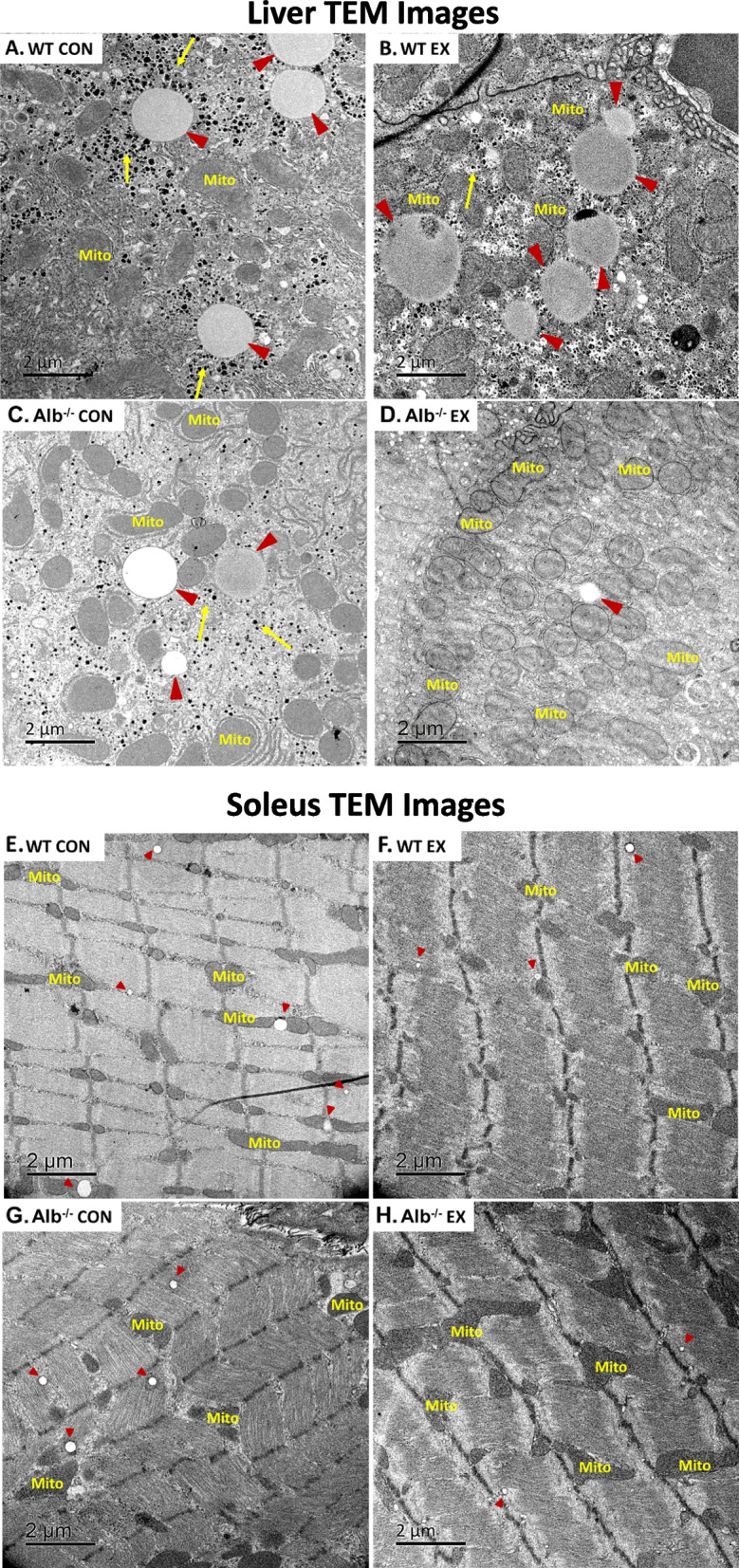
Representative transmission electron microscopy (TEM) images of liver and soleus. Magnification: X6000, scale bar: 2 μm**. A** Liver TEM images in WT control (CON). **B** Liver TEM images in WT exercise (EX). **C** Liver TEM images in Alb^−/−^ CON. **D** Liver TEM images in Alb^−/−^ EX. **E** Soleus TEM images in WT CON. **F** Soleus TEM images in WT EX. **G** Soleus TEM images in Alb^−/−^ CON. **H** Soleus TEM images in Alb^−/−^ EX. Mito: mitochondria; short red arrows point at lipid droplets; long yellow arrows point at glycogen granules

Representative TEM images of the liver and soleus in each group are shown in Fig. 5. As described above and shown in the images, Alb^−/−^ mice had smaller hepatic LDs than WT mice under sedentary conditions. Also, a lower area density of glycogen was observed in the Alb^−/−^ mice. In WT mice, LDs were more in proximity with glycogen, while in Alb^−/−^ mice they were in close contact with mitochondria under sedentary conditions. After exercise, the number and size of LDs were reduced in Alb^−/−^ mice but experienced augmentation in WT. The area density of glycogen reduced after exercise in both genotypes. In soleus, there was no difference in LD area density and size between genotypes. In Alb^−/−^ mice, LDs were less associated with mitochondria compared to WT. Glycogen in the soleus was too low to be detected in TEM images.

### Hepatic LDs in WT mice were maintained using carbon backbone potentially from glycogen during exercise

**Fig. 6:**
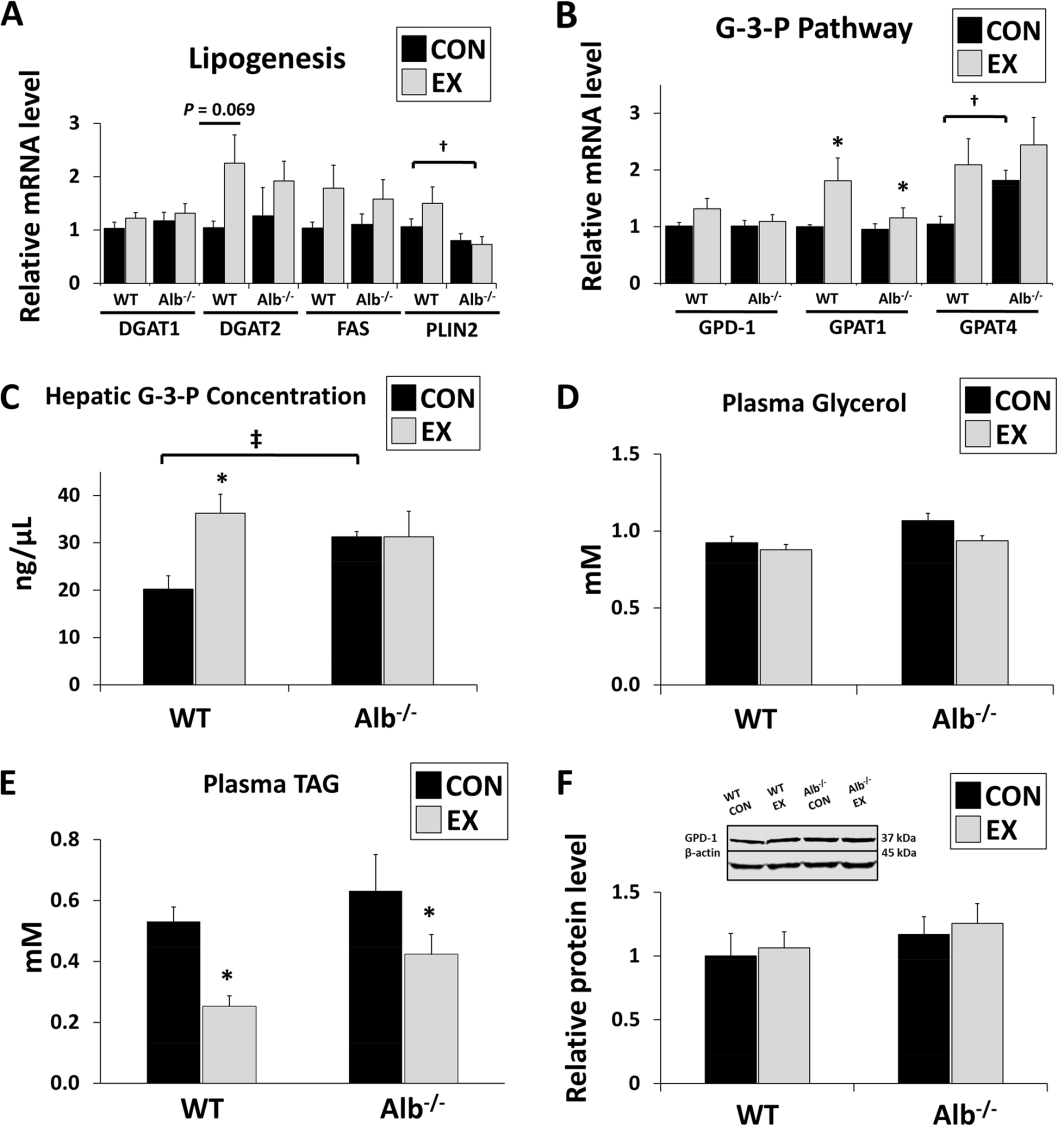
Large lipid droplets (LDs) in WT mice were maintained using carbon backbone potentially from glycogen during exercise **A**. Liver gene expression related to lipogenesis. DGAT1: Diacylglycerol O-acyltransferase 1; DGAT2: Diacylglycerol O-acyltransferase 2; FAS: Fatty acid synthase; PLIN2: Perilipin 2. **B** Liver gene expression related to G-3-P pathway. GPD-1: glycerol-3-phosphate dehydrogenase 1; GPAT1: Glycerol-3-phosphate acyltransferase 1; GPAT4: Glycerol-3-phosphate acyltransferase 4. **C** Hepatic G-3-P concentration. **D** Plasma glycerol concentration. **E** Plasma triacylglycerol (TAG) concentration. **F** GPD-1 protein expression. EX: exercise, CON: control. Analysis by ANOVA. *n* = 6 per group. Data are presented as the mean ± S.E.M. mRNA results normalized to 18S and protein results normalized to β-actin. Main effect of genotype, ^†^*P* < 0.01, ^‡^*P* < 0.05. EX different from CON within a genotype, * *P* < 0.05

The proximity between glycogen and LDs in WT mice led us to hypothesize that glycogen is involved in LD biogenesis, especially in response to acute exercise. Measurements were made regarding gene expression in lipogenesis and the G-3-P synthesis pathway. FAS is involved in de novo lipogenesis. DGAT1 and DGAT2 catalyze the conversion of diacylglycerol and fatty acyl CoA to TAG. PLIN2 promotes the stability and accumulation of LDs. DGAT2 mRNA expression showed an increasing trend in WT mice after exercise (Fig. 6A. *P* = 0.069). There was a main effect of genotype for PLIN2 expression with WT higher than Alb^−/−^ mice (*P* < 0.01). For genes involved in G-3-P pathway (Fig. 6B), GPD-1 is responsible for converting DHAP to G-3-P. GPAT1 and GPAT4 further incorporate G-3-P into lysophosphatidic acid, which will be later converted to diacylglycerol. GPAT1 expression increased significantly after exercise in both genotypes (main effect of exercise, *P* < 0.05). GPAT4 in Alb^−/−^ mice had higher expression than that WT (main effect of genotype, *P* < 0.01). The concentration of hepatic G-3-P, plasma TAG and glycerol was measured. For hepatic G-3-P concentration, there was a 1.5-fold elevation in WT mice after exercise but no alteration in Alb^−/−^ mice with exercise (Fig. 6C. *P* < 0.05). Plasma glycerol showed no significant difference in terms of genotype or exercise effect (Fig. 6D), but plasma TAG experienced a marked reduction after exercise in both groups (Fig. 6E main effect of exercise, *P* < 0.05). Moreover, in WT mice after exercise, there was a negative correlation between the percentage change in lipid and glycogen area density (R = -0.32, *P* < 0.05), suggesting glycogen was a potential supply for the lipid growth in WT mice. On the contrary, Alb^−/−^ mice showed no significant correlation between these two variables. No significant difference was found in GPD-1 protein expression among the groups (Fig. 6F).

### Reduction in Alb^−/−^ LDs is not associated with expression of β-oxidation genes

**Fig. 7:**
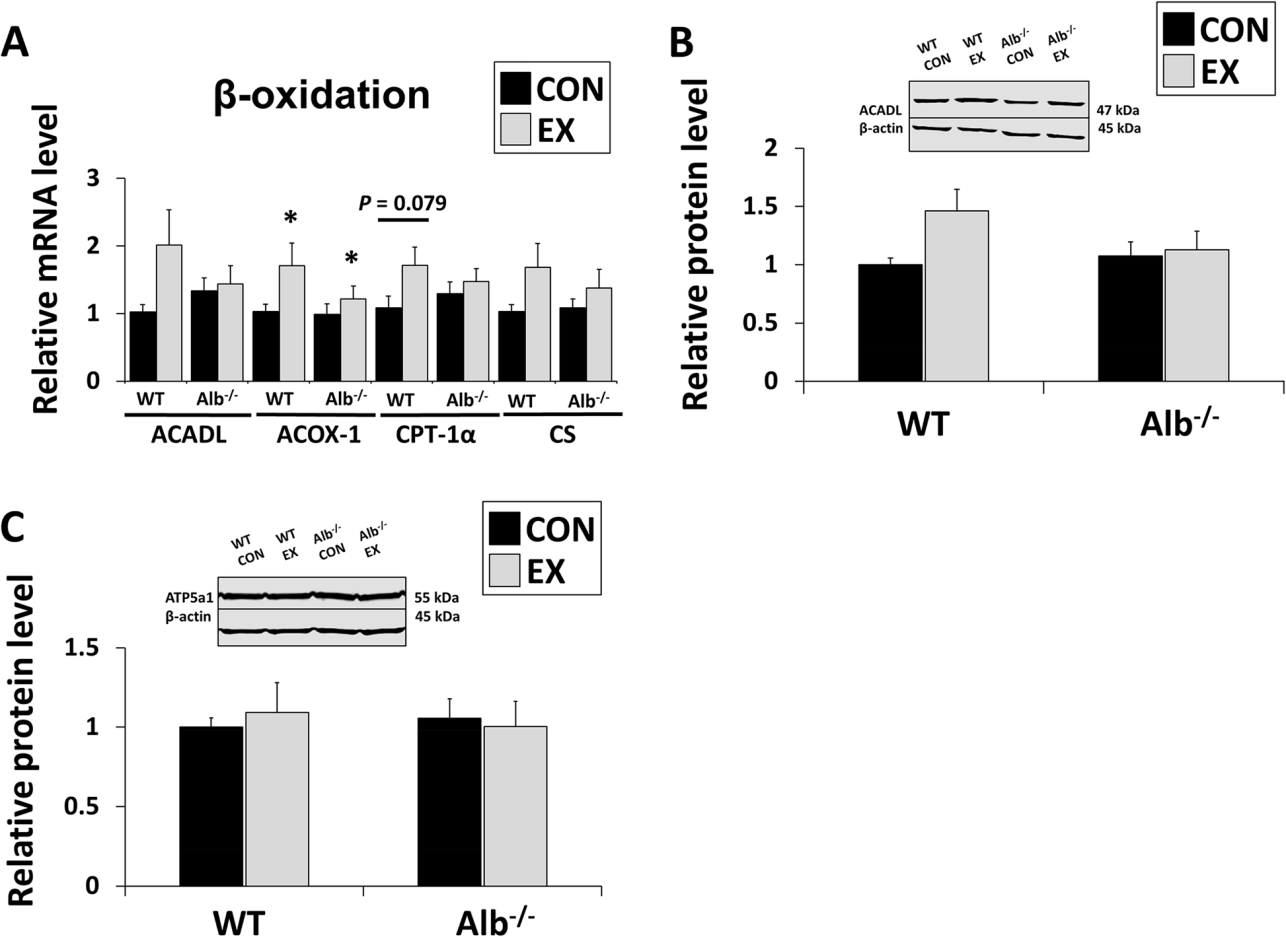
Reduction in Alb.^−/−^ lipid droplet (LD) is not associated with β-oxidation **A** Liver gene expression related to β-oxidation. ACADL: acyl-CoA dehydrogenase long chain; ACOX1: Acyl-CoA Oxidase 1; CPT1-α: Carnitine palmitoyltransferase-Iα; CS: Citrate synthase. **B** Hepatic ACADL protein expression. C) Hepatic ATP5a1 protein expression. ATP5a1: ATP synthase F1 subunit α. EX: exercise, CON: control. Analysis by ANOVA. *n* = 6 per group. Data are presented as the mean ± S.E.M. mRNA results normalized to 18S and protein results normalized to β-actin. EX different from CON within a genotype, * *P* < 0.05

To test in Alb^−/−^ mice if the reduction in LDs after exercise was associated with their β-oxidation capacity, the expression of genes involved in β-oxidation was measured, including CS, CPT-1α, ACADL and ACOX-1 (Fig. 7A). CS (a TCA cycle enzyme) is commonly used as a marker for mitochondria. CPT-1α facilitates the transport of long chain fatty acids into the mitochondria, and ACADL is an enzyme in the mitochondrial β-oxidation pathway. ACOX-1 is involved in peroxisomal β-oxidation. Although exercise elevated gene expression of ACOX-1 in both genotypes (main effect of exercise), there were no significant main effects of genotype or interactions that would suggest higher expression in Alb^−/−^. Protein expression was assessed for a β-oxidation gene (Fig. 7B. ACADL) and an electron transport chain-related gene (Fig. 7C. ATP synthase subunit ATP5a1). There was a non-significant trend for an increase in ACADL protein in WT mice after exercise (*P* = 0.059), but no significant differences or trends were observed in ATP5a1.

## Discussion

**Fig. 8:**
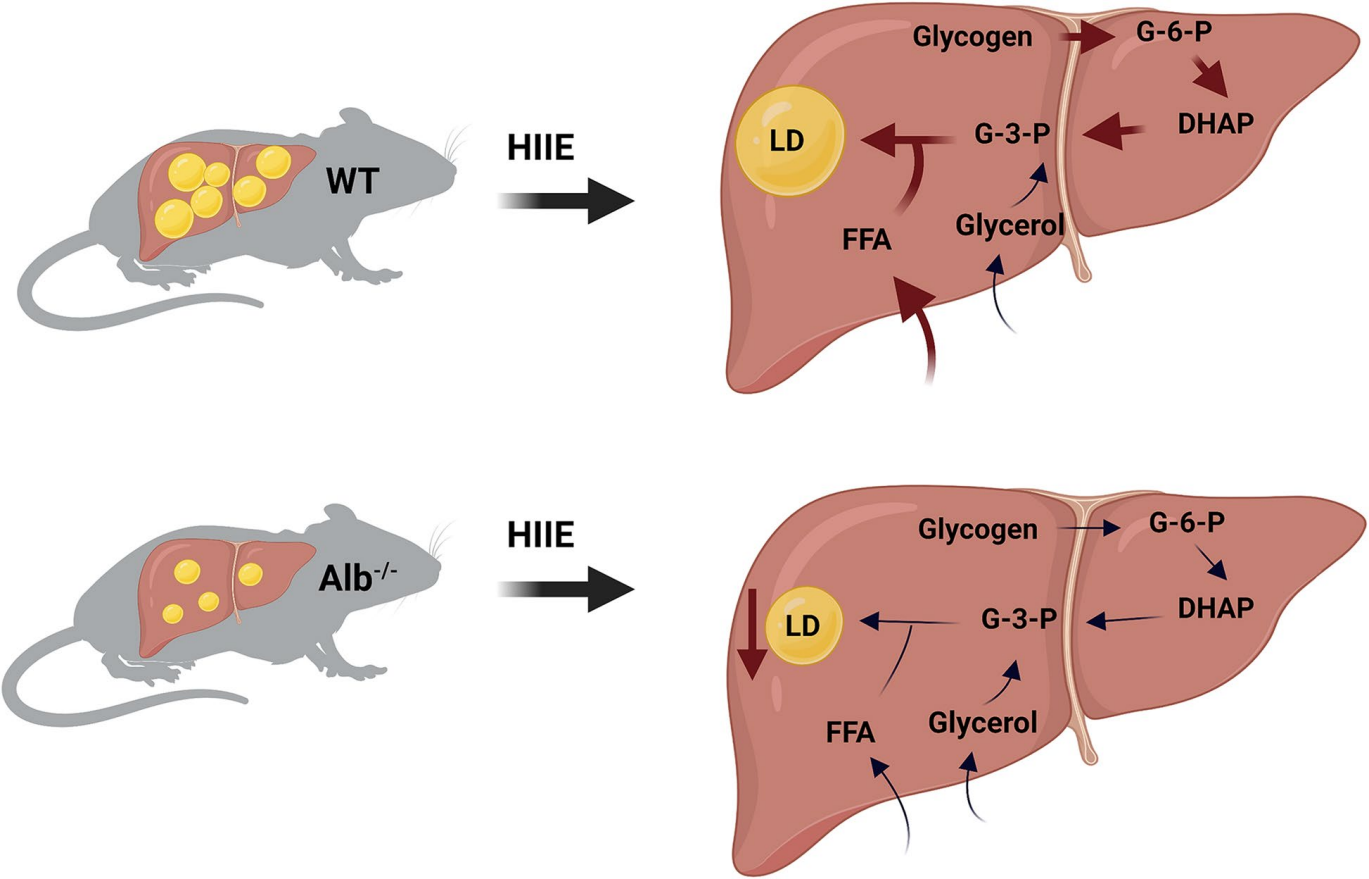
Graphical depiction of hepatic lipid droplet (LD) changes in mice after acute exercise. At rest, wildtype (WT) mice exhibited higher abundance and larger size in hepatic LDs while albumin knockout (Alb^−/−^) mice had lower LD abundance and smaller LD size. After a single bout of high-intensity interval exercise (HIIE), the hepatic LDs in WT mice maintained, alongside their plasma free fatty acid (FFA) levels that were higher than Alb^−/−^ mice and their exercise-induced elevation of hepatic glycerol-3-phosphate (G-3-P). FFA and G-3-P are recognized as the substrates for triacylglycerol synthesis, the core of LD. G-3-P elevation could be derived from hepatic glycogen. Conversely, we observed a reduction in LD size in response to exercise in Alb^−/−^ mice; unlike in WT mice, Alb^−/−^ mice did not experience an exercise-induced elevation of G-3-P in the liver, which was potentially due to their lower glycogen supply. The figure was created with BioRender.com

Here it was investigated how albumin deficiency, a condition known to alter metabolic health, would impact the body’s response to another health-promoting factor (challenging exercise). It was of interest to test the effects of each factor, alone and in combination, on hepatic LD abundance, sizes, and localization. The primary findings of the study are summarized graphically in Fig. 8. The results showed that sedentary Alb^−/−^ mice had lower abundance and size of hepatic LDs, which were particularly associated with mitochondria. WT mice exhibited higher abundance and larger size of hepatic LDs, which were surrounded by glycogen granules. In response to acute exercise, hepatic LDs in Alb^−/−^ mice showed a dramatic reduction in size, while WT hepatic LDs were maintained or exhibited a slightly elevated size. Substantially lower hepatic glycogen was observed in Alb^−/−^ than WT. The present report is the first to describe a metabolic and spatial relationship between hepatic glycogen and LD maintenance. The maintenance of hepatic LDs in WT mice at or slightly above sedentary levels after exercise was associated with elevated G-3-P level and higher glycogen-LD interaction, suggesting maintenance of LD size was potentially supported by glycogen use. Conversely, the reduction in hepatic LDs in Alb^−/−^ mice could be due to low hepatic glycogen supply. Alb^−/−^ mice exhibited an enhanced ability to reduce hepatic LD abundance in response to exercise. These findings indicate a combined effect of albumin deficiency and acute exercise in reducing ectopic lipid droplet accumulation in the liver. Contrarily, muscle tissue in Alb^−/−^ and WT tended to have a similar response to HIIE regarding LD abundance, number and its interaction with mitochondria. This might be a potential reason why Alb^−/−^ exhibited similar exercise capacity as WT mice. Overall, findings in the present study showed a unique response from the combination of albumin deficiency and HIIE in hepatic lipids, which may have implications for providing insights into NAFLD treatment.

The first striking finding in the present study is evidence for a potential role of glycogen in LD maintenance in the liver. The most widely accepted lipogenic pathway is that LDs are budding from ER, as DGATs needed for the last step of TAG synthesis reside on ER membrane [7]. Meanwhile, GPAT1 and GPAT2 are located in mitochondria, contributing to the first step of converting G-3-P to the final product of TAG [7]. Thus, there is an important relationship between LDs, the ER, and mitochondria. It is noteworthy that the LD maintenance during exercise in WT mouse liver led to more LD-mitochondria interactions, and this may have been a result of mitochondria’s participation in the TAG synthesis pathway. Nonetheless, the association of LDs and other cellular structures was also of interest, such as LD-glycogen interactions. Especially in the WT CON group, LDs were associated with glycogen granules. Moreover, after exercise, the reduction in glycogen in WT mice coincided with higher LD abundance. Despite the closely linked biological and structural hallmarks, the connection between glycogen and LDs is largely unappreciated in the literature. To date, only one published paper has reported glycogen’s role in LD biogenesis, and this metabolic relationship was shown in brown adipose tissue [28]. Here, it is proposed that glycogen granules can play a role in providing substrate to glycolysis which produces the G-3-P carbon backbone for TAG in hepatic LD assembly. The high glycogen utilization during exercise in WT mice indeed coincided with an elevation of G-3-P levels in the liver. Since G-3-P could also be derived from glycerol, the plasma glycerol level was assessed, but no significant change was found. Furthermore, glycerol is a marker of whole-body lipolysis. Thus, these data indicated that lipolysis may have proceeded normally in Alb^−/−^ despite their lower FFA mobilization into plasma. These data added to the evidence that the rising G-3-P after exercise was likely from DHAP (a glycolytic intermediate) rather than from glycerol. After exercise, Alb^−/−^ mice exhibited no change in G-3-P level, which corresponded to their declining LD content and size. These results indicated that in Alb^−/−^ mice during exercise, glycogen may be mostly used for glycolysis rather than for LD maintenance. Additionally, the consistently abundant G-3-P level potentially resulted from low FFA in Alb^−/−^ mice, leading to slower DHAP clearance into the TAG synthesis pathway. In agreement with our finding, ^14^C-labelled glucose [29] and ^13^C-glucose [30] label the glycerol backbone of TAG in the liver, demonstrating the use of glycolysis to provide the TAG backbone in the liver in vivo. Still, there are other hypotheses about the possible link between glycogen and LDs. For example, glycogenin was reported to have amphiphilic properties that make it strongly associated with phospholipids on the LD surfaces [31]. In this case, the LDs provide a harboring surface for glycogenin to grow into glycogen granules. Nevertheless, the biogenesis of LDs is complex and is not dependent solely on the pathways presented here. The focus of the present report is on the G-3-P pathway in LD biogenesis, given the fact that TAG is the main component of LDs and the addition of fatty acids to G-3-P backbone is the primary synthesizing pathway of TAG. Other pathways and lipids are also involved in LD biology and will be studied in future research. Overall, the overlaps in architecture and physiology between glycogen and LDs indicate an interesting but previously overlooked topic. Future studies should be conducted to reveal the incorporation of carbon from hepatic glycogen into LDs. It is worth noting that the features described above are potentially restricted to the liver as no significant change was found in LD size in muscle samples. Also, muscle glycogen is nearly 50 times lower than liver, making it difficult to observe LD-glycogen interaction under TEM.

Another central finding of the present study is the different sizes of hepatic LDs between WT and Alb^−/−^ mice. The small-sized hepatic LDs in Alb^−/−^ mice were associated with low plasma FFA supply. LD sizes have significant implications for their metabolic fates. It has been demonstrated that small LDs tend to stay constant in size, while large LDs have a propensity toward growing even larger [32]. The reason could be that small LDs lack an abundance of TAG synthesis enzymes while large LDs contain more of such enzymes. Large LDs provide a greater total surface area for more TAG synthesis enzymes to dock. Those TAG synthesis enzymes include GPATs, acylglycerol-3-phosphate acyltransferases and DGATs [8]. Another interesting perspective is that when large LDs were coated with more LD-related proteins, such as perilipins, their presence limits the access of ATGL and protects it from lipolysis [33, 34]. It is then understandable that PLIN2 was found to be upregulated in patients with NAFLD and its ablation improved hepatic steatosis [35]. In agreement, PLIN2 expression is also low in Alb^−/−^ mice alongside smaller LDs and less hepatic TAG. If hepatic LDs can initially limit their sizes upon generation or in response to a lifestyle factor such as exercise, the likelihood of unrestricted expansion in lipid contents may be diminished. As LDs provide protection from lipotoxicity and mitochondrial damage, the interfering with LD formation in the presence of a high plasma FFA concentration might potentially lead to reduced buffering of FFA and other lipid species, increasing risk for lipotoxicity and oxidative stress. However, in the case of Alb^−/−^ model, the formation of LDs is reduced by interrupting the FFA uptake into the liver. Therefore, it would be reasonable to hypothesize that reduced albumin and FFA in circulation may protect from lipotoxicity, as discussed previously [17, 36]. Further work on the importance of balancing LD biogenesis with the plasma FFA supply is an important area of future investigation. Indeed, Alb^−/−^ had smaller LDs and then a particularly favorable response to exercise. However, it was noted that the results for total LD content and TAG content differed somewhat. In Alb^−/−^ mice, apparent LD content in the liver was reduced by exercise based upon TEM image analysis, but this was not apparent based upon biochemical analysis of liver TAG. One reason for this discrepancy between LD and TAG measurement could be that when the average LD diameter dropped substantially during exercise in Alb^−/−^ mice, this led to the development of very small LDs that were not identifiable in TEM images. Another possibility is that TAG residing outside LDs rose during exercise. Finally, as described for skeletal muscle analysis [37], it is possible that in liver TEM image analysis is inherently more accurate than biochemical analyses. Also, it is important to note that the LD sizes presented were averages from a population of LDs within each tissue. It is unknown whether LDs in any certain size class were metabolized differently from those in other size ranges, but it is clear that the overall size distributions shift with exercise. The consumption rate of LDs in various size ranges would be methodologically challenging but is worthy of further investigation. Nonetheless, despite some remaining uncertainly about the total neutral lipid storage in the liver, a robust and fundamental finding of the present study is that the average LD size was lower in Alb^−/−^ than WT and that Alb^−/−^ mice showed a striking further reduction in LD size in response to exercise.

In Alb^−/−^ mice, which had small LDs under sedentary conditions, the small LDs were further reduced in size in response to exercise. This could be attributable to the fact that smaller LDs have a higher surface-area-to-volume ratio, making them more available for metabolic reactions [38, 39]. Here, it was initially hypothesized that β-oxidation was the reason for shrinking LDs given the close contact between LDs and mitochondria in Alb^−/−^ mice. However, there was no statistical difference in the β-oxidation genes or proteins tested. Nonetheless, it is noted that increased flux could hypothetically occur without changes in gene or protein expression. It is also noted that a recently published paper emphasized the significance of β-oxidation in LD-associated mitochondria in the liver [40]. A four-week exercise training increased the LD-mitochondrial interaction in muscle in mice [41] due to the increased size of mitochondria. Meanwhile, growing evidence has supported the idea of lipophagy as a possible explanation for the reduction in hepatic LDs [42, 43]. It was not possible to confirm this idea, as no significant abundance of autophagosomes was observed under TEM. Overall, it is still unclear whether the main factor in hepatic lipid responses to exercise is the regulation of LD storage, LD utilization, or lipophagy. Nonetheless, modulating each of these factors is a potential avenue for improving liver health. The responses of hepatic lipid to exercise in the present study were not a result of any exercise-induced changes in plasma FFA concentration because the mice were challenged with an exercise type that did not raise plasma FFA concentration. Unlike low-intensity exercise, higher exercise intensities do not raise plasma FFA [44], yet they still have potent impacts on metabolism in other ways. It is possible that the present findings for responses to HIIE are different from that which would occur in response to a lower-intensity exercise type. Nevertheless, HIIE was employed in this paper as it is expected to have various health benefits, has grown in popularity over the years, and was reported to be more enjoyable than lower-intensity exercise [45].

Alb^−/−^ mice display low hepatic LD content and glycogen, indicating that this could be an interesting model for elucidating the dynamics of hepatic metabolism. The lessons learned could be relevant to NAFLD, and that potential relevance could be confirmed by future research. The reduction in plasma FFA leads to lower supply of FFA for esterification to G-3-P, hence lowering the hepatic LD content. It should be noted that the reduction of plasma FFA in Alb^−/−^ mice is even more pronounced when the fasting duration is longer [17] and thus the blunting of FFA supply to the liver can be quite substantial at times of day when animals are fully postabsorptive. The present finding of low LD content in Alb^−/−^ liver is in line with a previous study where Alb^−/−^ mice manifested alleviated hepatic steatosis from obesity induced by a high fat diet [36]. It should also be noted that in human subjects with a genetic disorder called congenital analbuminemia (CAA), no or very low albumin level is detected in plasma [46]. These patients can lead a normal life and exhibit some modest changes in the expression of other proteins in plasma. Although plasma proteins like globulins are elevated in patients with CAA, the overall protein level is not well compensated, such that the total plasma protein concentration is lower than normal [46]. This agrees with the findings on Alb^−/−^ mice [17]. It is still unknown why patients with CAA and Alb^−/−^ mice can survive in the absence of the most highly abundant plasma protein. Future work on this mouse model could ultimately lead to discoveries that may be translated into therapeutics to enhance health. While it would not be desirable to reduce albumin expression in patients, it may be a reasonable goal to develop potential therapeutics that would alter specific functions of albumin, such as FFA transport.

### Study strengths and limitations

The strengths in the present study are the combined utilization of TEM and biochemical assays, which allowed a comprehensive and direct investigation of LD localization, size and morphology. This led to the key finding of the association between LD and glycogen granules in the liver. The size distribution of LD was also derived from the direct measurement under TEM. The study has some limitations including the sole investigation on young male mice. Also, the focus was on a single LD biogenesis pathway rather than testing all theoretically relevant pathways. Future research on female mice, mice at different ages, and other signaling pathways are to be conducted. 

## Conclusions

In summary, in this paper it was shown that Alb^−/−^ mice possessed smaller hepatic LDs. Additionally, this mouse model manifested an enhanced capacity to reduce hepatic LDs in response to exercise. In an attempt to reduce hepatic lipids, benefits brought by exercise nowadays are far from satisfactory, and the present findings indicate that FFA, albumin, or specifically the FFA-albumin interaction may restrain or delay the effects of exercise on the liver in WT animals. Moreover, an important potential role of hepatic glycogen in the LD biogenesis was discovered. The findings illustrate a combined effect of albumin deficiency and an exercise bout for reducing ectopic lipids and LD size in the liver.

## Data Availability

Data will be available from the corresponding author upon reasonable request.
